# Multiple Thrombotic, Infectious, and Cardiopulmonary Complications Following Laparoscopic Converted to Open Colectomy Procedure: A Case Report and Literature Review

**DOI:** 10.7759/cureus.49384

**Published:** 2023-11-25

**Authors:** Kateryna Georgiyeva, Brian G Nudelman, Harendra Kumar, Shiv Krishnaswamy, Juliana Cazzaniga

**Affiliations:** 1 Internal Medicine, Memorial Healthcare System, Pembroke Pines, USA; 2 Medicine and Surgery, Dow University of Health Sciences, Karachi, PAK; 3 Medical School, Florida International University, Herbert Wertheim College of Medicine, Miami, USA

**Keywords:** post-colectomy abscess, hypercoagulable state, surgical complications, colorectal adenocarcinoma, superior mesenteric vein thrombosis

## Abstract

All surgeries, from minor procedures, such as sutures, to major surgeries, such as open abdominal surgery, carry with them risk for complications. Among the most frequently encountered complications are surgical site infections and thrombotic complications. Less frequently, cardiac complications such as atrial fibrillation are seen. In this case report, we discuss the various complications encountered during the hospital stay of a 61-year-old male following a laparoscopic converted to open colectomy procedure for the treatment of a colorectal mass. Following surgery, a surgical pathology report revealed a newly diagnosed stage 3b colorectal adenocarcinoma. Multiple abscesses in the abdominopelvic cavity were discovered on computed tomography (CT), revealing a major surgical site infectious process. These findings warranted emergent surgical intervention and placement of multiple Jackson-Pratt drains. Due to previously untreated carcinoma promoting a prothrombotic state, the patient developed numerous thrombotic complications such as segmental pulmonary embolism, superior mesenteric vein thrombosis, and superficial thrombophlebitis of the saphenous veins. He also developed new-onset paroxysmal atrial fibrillation secondary to postoperative pain, as well as bilateral pleural effusions. Here, we shed light on the mechanisms of development of such complications, as well as the management and methods for prevention.

## Introduction

Post-surgical complications are unfortunate and very common problems that can occur even after the most minor operations. Especially in colorectal surgery, there is a 28% chance of developing at least one of these complications [1]. The most common complications of colorectal surgery are directly related to the site of surgery; these include infections, gastrointestinal (GI) issues such as ileus and obstruction, surgical site hematomas, and wound dehiscence [2]. Although the majority of post-surgical issues are associated with the colon, colectomies can also cause significant bleeding, genitourinary problems, thromboembolisms, and other cardiopulmonary complications such as pneumonia or ventilator dependence [3]. Several risk factors for developing the mentioned surgical complications have been identified in literature. History of strokes, coronary artery disease, myocardial infarction, heart failure, as well as old age, ascites, hypoalbuminemia, and hypernatremia have all been shown to increase the risk of postoperative complications in colorectal surgery patients [1,2]. The presence of multiple post-surgical complications has been associated with increased hospital length of stay as well as increased mortality in these patients. A study by Longo et al. showed that postoperative ileus is associated with the development of additional post-surgical complications in almost 60% of non-emergent colectomy patients [4]. Furthermore, a correlation has been appreciated between ileus and the development of post-colectomy deep vein thrombosis (DVT) [5]. We present a case of a patient who presented status post right colectomy who developed sepsis secondary to the formation of multiple intra-abdominal abscesses as well as DVT and new-onset atrial fibrillation (Afib).

## Case presentation

This case report describes the hospital course of our 61-year-old male patient with a past medical history of newly diagnosed colon adenocarcinoma stage 3b with mesenteric margin, status post right colectomy, and end ileostomy of the right lower quadrant performed two weeks prior to presentation. He presented to the emergency department (ED) with generalized weakness and fatigue, along with fever 102°F about two weeks after the procedure. The patient described that in the two weeks following his original procedure, he slowly felt progressive malaise and fatigue. He was also found to have intermittent, mild, dull lower abdominal pain and mild dysuria. Shortly prior to presenting to our hospital, the patient visited the ED at another hospital for the same symptoms and had a computed tomography (CT) abdomen/pelvis done which revealed multiple fluid-filled collections with the largest being over the left subphrenic space concerning for an intra-abdominal abscess. The patient was found to be septic in the ED, with a heart rate of 120, a respiratory rate of 30, a temperature of 38.5, and a white blood cell count of 14.4, with the likely source of infection being intra-abdominal abscess. He was started on intravenous (IV) piperacillin-tazobactam for empiric coverage of intra-abdominal infection and was admitted to the hospital for further observation and workup.

**Figure 1:**
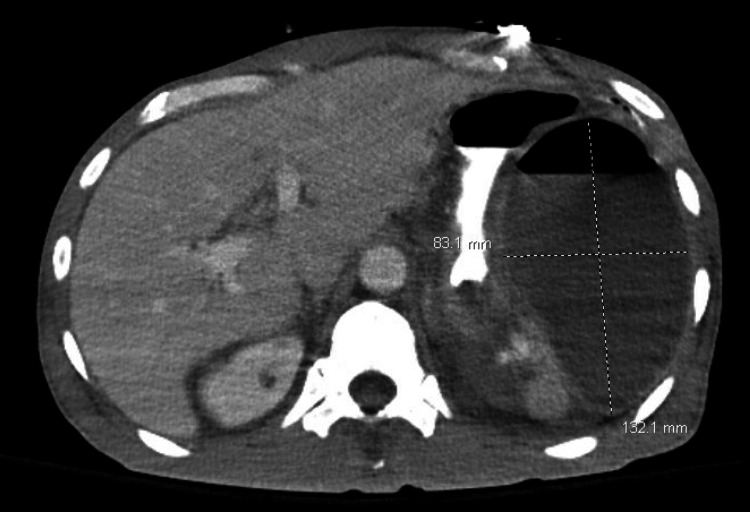
A computed tomography scan of the patient's abdomen in a sagittal view. The perisplenic abscess is marked using white lines.

A CT chest with IV contrast was done to rule out any potential metastasis of the colorectal adenocarcinoma to the lungs. The results of the CT revealed no evidence of pulmonary embolism (PE), yet small bilateral pleural effusions, left more than right, were found. An echocardiogram revealed grade 1 diastolic dysfunction with a hyperdynamic ventricular function. A CT abdomen/pelvis with oral and IV contrast showed four major encapsulated collections of fluid throughout the abdomen concerning for abscess. These collections of fluid included the following: 1) perisplenic collection of fluid and gas measuring 13 x 8 cm, 2) fluid collection and gas measuring 6.5 x 5 cm inferior to the body of the pancreas, 3) fluid collection between the bladder and rectum measuring 9.7 x 7.1 cm, and 4) fluid collection measuring 4.4 x 1.8 cm surrounding the right lower quadrant ostomy. Figure 1 shows the biggest post-surgical abscess, adjacent to the spleen.

A CT-guided drainage of 2/4 of these fluid collections was done by interventional radiology the following day. Acid-fast Bacilli (AFB) smear and culture, fungal cultures, and bacterial Gram stain and cultures were taken from the drained fluid. These cultures were taken from the left upper quadrant as a result of the perisplenic abscess drainage and from the lower abdomen as a result of the drainage of the abscess between the bladder and rectum. Two Jackson-Pratt (JP) drains were placed by the surgery team, with one drain placed in the left upper quadrant, draining frank purulent fluid, and the other in the buttock area with serosanguinous drainage. Cultures taken from the fluid resulted in a couple days, with both drained abscess locations growing *Escherichia coli*. The patient was continued on IV piperacillin-tazobactam while sensitivities were pending. Results of culture sensitivities showed resistance of the *E. coli* bacterium to piperacillin-tazobactam, yet both strains remained susceptible to second- and third-generation cephalosporins. Possible mechanism of resistance to piperacillin-tazobactam was presumed to be the hyperexpression of narrow-spectrum beta-lactamase. Antibiotic therapy was changed to ceftriaxone, a third-generation cephalosporin, and metronidazole was added for increased anaerobic coverage.

The patient continued to improve on this therapy, yet another concern became prevalent. Due to pain, the patient converted from normal sinus rhythm to new-onset paroxysmal Afib with a rate of 130-160 beats per minute on telemetry monitoring. The patient's blood pressure at this time was 130/80, and he was saturating well on 5 L nasal cannula. IV fluid bolus was given, and he also received 5 mg metoprolol IV and digoxin 250 mcg IV. Following the administration of these agents, the patient converted back to normal sinus rhythm with a heart rate of 90-100. Transthoracic echocardiogram was done without structural abnormalities.

Due to continued labored breathing and oxygen (O2) requirements of 5 L nasal cannula to maintain adequate saturation, a chest X-ray was ordered. Results revealed bilateral pleural effusions, prompting thoracentesis and pleural fluid analysis in the setting of malignancy and infectious process. Pleural fluid analysis supported the diagnosis of a simple transudative pleural effusion, and culture of the pleural fluid did not reveal growth of any organisms.

Supplemental O2 was continued to maintain oxygen saturation (SPO2) greater than 92%. The patient's O2 requirements decreased to 4 L the following day. Pelvic JP drain was also removed at this time. Up to this point in the hospitalization, the patient had been bed-bound. New complaint of bilateral calf pain was noted. A lower extremity Doppler ultrasound revealed bilateral saphenous vein thrombi. This resulted in an anticoagulation switch full dose of enoxaparin.

Tachypnea with a respiratory rate of 30 and blood SPO2 in the low 90% was noted overnight. A CT with PE detecting protocol was ordered and revealed a small nonocclusive thrombus within the right middle lobe lateral segment. Atelectatic changes were also noted, and incentive spirometer use was encouraged. Pelvic JP drain was removed in the morning. The patient continued to receive full-dose enoxaparin anticoagulation, and O2 requirements improved significantly in the course of days. 

**Figure 2:**
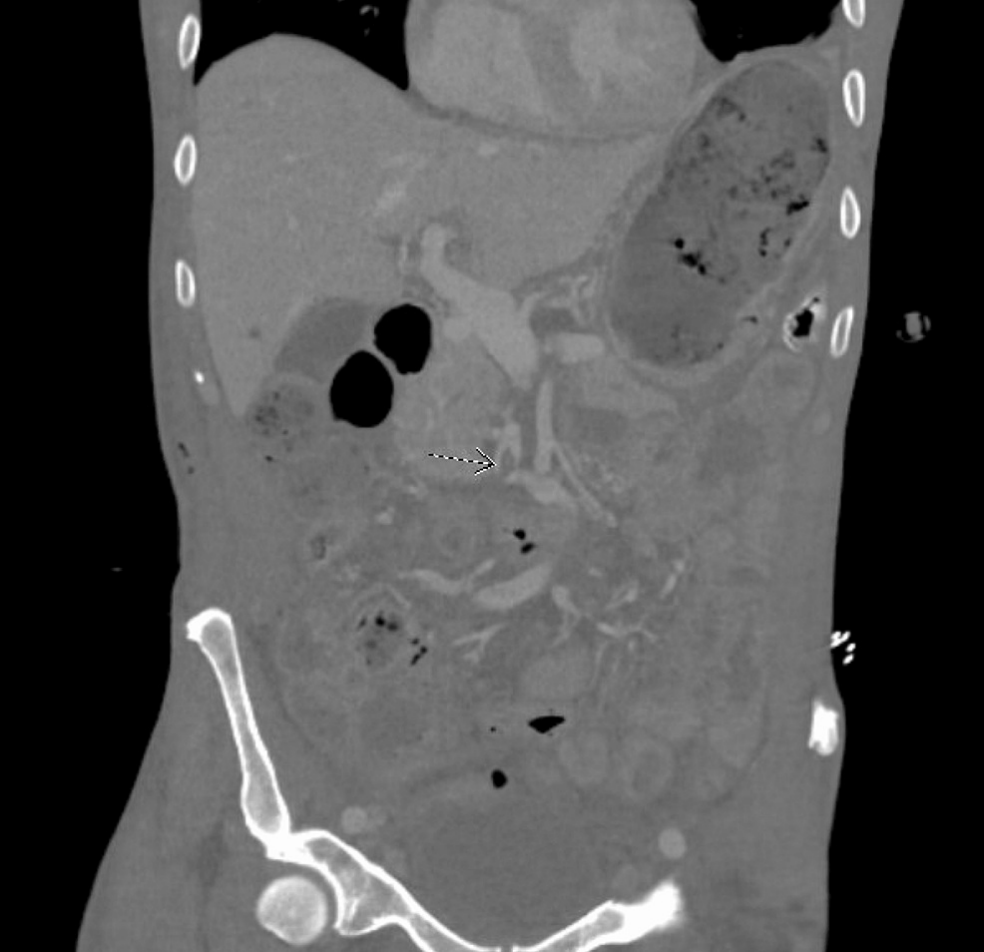
A computed tomography scan of the patient's abdominopelvic area in a coronal view, with an arrow pointing to the site of the superior mesenteric vein thrombosis.

Prior to discharge, a CT abdomen and pelvis with IV contrast was repeated. A decrease in the size of intra-abdominal abscesses was seen. A finding of near-complete narrowing of the distal superior mesenteric vein and nonocclusive thrombus proximal to the site of narrowing was seen. Figure 2 shows a CT scan of the abdominopelvic cavity in coronal view, with an arrow pointing to the site of the thrombus in the superior mesenteric vein. It was determined that this thrombus did not pose a severe threat and was also treated with full-dose enoxaparin anticoagulation.

The patient was ultimately discharged home with apixaban anticoagulation and follow-up with oncology for colorectal adenocarcinoma. IV antibiotic therapy was completed using a home health service through a midline.

## Discussion

This case report involves a 61-year-old male patient who underwent laparoscopic conversion to open colectomy surgery. It reveals a multifaceted and intricate web of medical complexities, necessitating a comprehensive and interdisciplinary approach to patient care. This case underscores the critical significance of adopting a holistic perspective in managing patients, encompassing various facets of postoperative care, including infectious, thrombotic, cardiac, and pulmonary complications.

In this case, the patient's postoperative course was marred by the development of multiple intra-abdominal abscesses following the colectomy procedure. These abscesses were distributed across several abdominal regions, including the perisplenic area, the space inferior to the pancreas, the region between the bladder and rectum, and the vicinity of the right lower quadrant ostomy. This intricate clinical scenario presented a considerable challenge, as intra-abdominal abscesses have the potential to rapidly progress to sepsis, a life-threatening condition. The patient exhibited clinical indicators of sepsis, including an elevated heart rate, respiratory rate, fever, and an increased white blood cell count, signifying the presence of a severe systemic infection that demanded immediate intervention.

The occurrence of multiple complications following laparoscopic conversion to open surgery is a notable and intricate clinical scenario that necessitates meticulous consideration [6]. These complications encompass infectious, thrombotic, cardiac, and pulmonary issues, thereby emphasizing the intricacy of postoperative care [6]. Beyond their impact on individual cases, the significance of these complications extends to broader implications for the surgical and healthcare domains. Patients and their families need to be well informed about the potential risks associated with surgical procedures, acknowledging the possibility of complications and the measures taken to mitigate such risks. The presence of multiple complications can substantially extend a patient's recovery duration. Complications like infections and thrombotic events can lead to prolonged hospital stays and increased patient discomfort, thereby necessitating proactive measures, including preventive antibiotics, anticoagulants, and early mobilization [6]. These cases can also serve as catalysts for quality improvement initiatives, inspiring further research into the prevention and management of postoperative complications. 

Radiological imaging, notably the CT scan of the abdomen and pelvis, played a pivotal role in diagnosing the intra-abdominal abscesses. The findings from these scans guided the subsequent treatment, underscoring the significance of advanced imaging techniques in identifying and managing postoperative complications.

In the management of infectious complications following surgery, including laparoscopic conversion to open surgery, the understanding of microbiological findings and antibiotic therapy assumes paramount importance [7]. Precise identification of the causative microorganisms through microbiological analyses, including cultures and sensitivity testing, is imperative to tailor antibiotic therapy effectively [7]. Timely and appropriate antibiotic administration is vital in addressing surgical site infections and other infectious complications, preventing their escalation, and reducing the risk of systemic infection and sepsis [7]. The choice of antibiotics should consider the spectrum of coverage needed, potential drug interactions, and the patient's medical history, including known allergies [7]. A judicious antibiotic regimen, guided by microbiological findings, stands as a cornerstone of postoperative care, facilitating the resolution of infections and promoting a successful recovery.

The management of infectious complications in this case involved CT-guided drainage of the abscesses by interventional radiology. This procedure precisely targeted the abscesses, enabling the evacuation of infected material and promoting recovery. The patient also received empiric antibiotic therapy with piperacillin-tazobactam, designed to offer broad-spectrum coverage of potential pathogens. Cultures obtained from the drained abscesses revealed the presence of *E. coli*, a common causative agent in intra-abdominal infections. The choice of antibiotics was adjusted based on the sensitivity results, with *E. coli* demonstrating resistance to piperacillin-tazobactam. Subsequently, the patient's treatment plan was modified to incorporate ceftriaxone, a third-generation cephalosporin, with the addition of metronidazole for enhanced anaerobic coverage.

The thrombotic complications observed in this case encompassed a segmental PE, superior mesenteric vein thrombosis, and bilateral saphenous vein thrombosis. These complications were further exacerbated by the patient's underlying colorectal adenocarcinoma, which created a prothrombotic state. Colorectal cancer is well documented in the medical literature as being associated with an elevated risk of thrombotic events, primarily due to the release of procoagulant factors and activation of the coagulation cascade [8]. The patient's medical history and the prothrombotic state associated with his cancer diagnosis played significant roles in the development of these complications.

The diagnosis and management of the thrombotic events entailed a combination of imaging studies and anticoagulation therapy. The segmental PE was identified through a CT PE protocol. Anticoagulation therapy was promptly initiated to impede further thrombus propagation and reduce the risk of complications related to PE. The patient's diagnosis of superior mesenteric vein thrombosis was primarily based on a CT scan of the abdominopelvic cavity, which disclosed near-complete narrowing of the distal superior mesenteric vein and a nonocclusive thrombus proximal to the site of narrowing. This thrombus was determined to pose a limited threat and was managed with full-dose enoxaparin anticoagulation. The presence of bilateral saphenous vein thrombosis was confirmed via a lower extremity Doppler ultrasound, prompting a transition to full-dose enoxaparin anticoagulation.

Several factors contribute to the development of Afib in postoperative patients. Surgical stress, pain, and inflammation can precipitate arrhythmias, particularly in patients with preexisting cardiac conditions [8]. Electrolyte imbalances and fluid shifts during surgery also play a role in the onset of postoperative Afib, underscoring the importance of vigilant monitoring and management to mitigate these risks [8]. In the context of this case, cardiac complications materialized as new-onset paroxysmal Afib. Postoperative pain emerged as a contributing factor, culminating in an episode of Afib with a heart rate fluctuating between 130 and 160 beats per minute. The management of this cardiac complication entailed the administration of an IV fluid bolus and specific medications, including metoprolol and digoxin, which effectively restored the patient to a normal sinus rhythm. Subsequently, a transthoracic echocardiogram was performed, revealing no structural abnormalities. These interventions played a pivotal role in addressing cardiac complications and ensuring cardiovascular stability.

The patient also presented with bilateral pleural effusions, which were detected through a chest X-ray. These effusions raised concerns, especially in the context of malignancy and infectious processes. A diagnostic thoracentesis was performed, and the pleural fluid analysis supported the diagnosis of a simple transudative pleural effusion. Importantly, no organisms were isolated from the culture of the pleural fluid. O2 therapy was initiated to maintain adequate SPO2, with the patient's O2 requirements progressively diminishing throughout the course of treatment. Additionally, the pelvic JP drain was removed, contributing to the overall improvement in the patient's condition.

Pulmonary complications in postoperative settings, such as the detection of bilateral pleural effusions, hold significance due to their potential implications in cases involving malignancy and infection [7]. The diagnostic procedures and findings obtained from pleural fluid analysis are crucial in understanding the underlying causes and guiding treatment decisions [7]. The progression of O2 requirements in response to pleural effusions necessitates careful monitoring, while the incorporation of incentive spirometer use contributes to respiratory recovery. Ultimately, the outcome of pleural effusion treatment directly impacts the patient's respiratory health and overall postoperative recovery.

The patient's susceptibility to postoperative complications was significantly influenced by various factors, including preexisting medical conditions and the presence of colorectal adenocarcinoma. The identified risk factors encompassed a history of strokes, coronary artery disease, and other medical conditions. Moreover, a correlation was observed between postoperative ileus and the development of DVT, further emphasizing the importance of managing postoperative ileus in colorectal surgery patients to prevent additional complications.

Surgical procedures, even when executed with the utmost precision, inherently entail the risk of postoperative complications. The identification of risk factors associated with these complications stands as a crucial endeavor for healthcare providers, enabling the anticipation and mitigation of potential issues. Several factors have been documented in the medical literature as contributing to a higher likelihood of postoperative complications [1,2,4]. These risk factors encompass age, medical history, surgical complexity, body mass index (BMI), smoking, substance abuse, nutritional status, and preoperative medications [7,8].

Age constitutes a pivotal factor, as advanced age is frequently correlated with an elevated risk of complications [8]. Older patients may exhibit reduced physiological reserves and an increased vulnerability to infections, cardiovascular events, and other postoperative issues. The patient's medical history can have a substantial impact on postoperative outcomes [9]. Conditions such as diabetes, heart disease, respiratory disorders, and autoimmune diseases can significantly heighten the risk of complications [8,9].

The complexity of the surgical procedure itself can influence the risk profile. More extensive surgeries, such as major abdominal procedures or organ transplants, inherently carry higher risk factors [10]. BMI emerges as another factor that can sway the likelihood of complications. Both obesity and being underweight can increase the risk of complications. Obesity is linked to wound healing challenges, while underweight individuals may have limited physiological reserves [7-9].

Patients who smoke or engage in substance abuse may be confronted with a heightened risk of complications, including difficulties in wound healing and an augmented risk of cardiac events [2,4]. Additionally, a patient's nutritional status assumes critical significance. Malnutrition or preoperative weight loss can compromise a patient's capacity to heal and recover following surgery [5]. Adequate nutrition is indispensable for effective tissue repair. Furthermore, preoperative medications, such as anticoagulants and steroids, can exert significant influences on postoperative outcomes, necessitating a thorough evaluation of their potential impact on bleeding tendencies and the body's stress response [5,8].

Postoperative ileus and DVT stand as two distinct complications that can materialize after surgery. However, an observed correlation between these complications underscores the need for healthcare providers to remain vigilant about potential interconnections [11]. Postoperative ileus, characterized by a temporary disturbance in normal bowel function, can result in various issues, including dehydration and immobility. These factors, in turn, elevate the risk of DVT [11]. Patients in the recovery phase following surgery often experience postoperative ileus, during which their bowel movements become sluggish or temporarily cease [11]. This condition can lead to dehydration, as patients may find themselves unable or reluctant to consume fluids and food [12]. Dehydration is a recognized risk factor for the development of DVT, as it promotes the formation of blood clots [10,11]. Furthermore, the immobility resulting from the discomfort associated with postoperative ileus can further enhance the risk of DVT. Prolonged periods of immobility can result in the pooling of blood in the lower extremities, increasing the likelihood of clot formation [12].

While postoperative ileus and DVT are separate complications, healthcare providers must remain cognizant of the potential correlation between these intricate clinical entities. The development of infectious, thrombotic, cardiac, and pulmonary complications in this case was driven by a complex interplay of factors. The patient's colorectal adenocarcinoma and the prothrombotic state it induced were key drivers behind the thrombotic complications. Furthermore, the patient's surgical history and preexisting medical conditions created a vulnerable clinical context for the development of these complications.

The management of these complications necessitated a multidisciplinary approach, emphasizing the pivotal role of various healthcare specialists in safeguarding the patient's well-being. Effective management strategies were meticulously employed for each specific complication, encompassing radiologically guided drainage, anticoagulation therapy, and medications to address cardiac arrhythmias. Regarding prevention, a thorough understanding of risk factors and patient-specific vulnerabilities remained essential in mitigating the likelihood of complications in colorectal surgery patients.

The case presented here exemplifies the intricate nature of postoperative complications following colorectal surgery. It underscores the importance of early detection, prompt intervention, and a coordinated, multidisciplinary approach to care. This discussion serves not only as a means of dissecting the complexities of this case but also as a wellspring of insights for healthcare providers who may encounter similar challenges in managing postoperative complications in colorectal surgery patients. Furthermore, it emphasizes the need for further research and studies to enhance our understanding of these complications and improve patient care and outcomes in the future.

## Conclusions

This case report highlights a rare clinical scenario of a post-colectomy abscess further complicated by the development of new-onset Afib, PE, and superior mesenteric vein thrombosis. The patient's management required a comprehensive approach, including surgical intervention to address the abscess, cardiology's management of the Afib, and management of the DVT by the medicine team. Despite the complexities, the patient was successfully treated through prompt diagnosis and evidence-based management of each of his problems. This case underscores the importance of early recognition and timely management of common complications in postoperative patients, as well as the need for vigilance toward the development of rare complications in high-risk populations. Possible interventions to decrease potential implications for future clinical practice may include adjustment of hospital protocols to achieve greater postoperative infection control. Further research and clinical experience will be essential to identifying optimal preventative strategies to reduce complications in postoperative patients.
